# Laparoscopic Bullet Removal in a Penetrating Abdominal Gunshot

**DOI:** 10.1155/2016/2712439

**Published:** 2016-07-25

**Authors:** Christos Stefanou, Nicolaos Zikos, George Pappas-Gogos, Spyridon Koulas, Ioannis Tsimoyiannis

**Affiliations:** ^1^Department of Surgery, “G. Hatzikosta” General Hospital, Makriyianni Avenue, 45001 Ioannina, Greece; ^2^Department of Surgery, Filiates General Hospital, 1 Mpempi Street, 45600 Filiates, Greece

## Abstract

Penetrating abdominal trauma has been traditionally treated by exploratory laparotomy. Nowadays laparoscopy has become an accepted practice in hemodynamically stable patient without signs of peritonitis. We report a case of a lower anterior abdominal gunshot patient treated laparoscopically. A 32-year-old male presented to the Emergency Department with complaint of gunshot penetrating injury at left lower anterior abdominal wall. The patient had no symptoms or obvious bleeding and was vitally stable. On examination we identified 1 cm diameter entry wound at the left lower abdominal wall. The imaging studies showed the bullet in the peritoneal cavity but no injured intraperitoneal and retroperitoneal viscera. We decided to remove the bullet laparoscopically. Twenty-four hours after the intervention the patient was discharged. The decision for managing gunshot patients should be based on clinical and diagnostic findings. Anterior abdominal injuries in a stable patient without other health problems can be managed laparoscopically.

## 1. Introduction

Since the time of World War I, selective nonoperative management (NOM) has become more widely accepted and negative laparotomies have been decreased. Laparoscopy for penetrating trauma has been described since the 60's, as a method to minimize unnecessary laparotomies [1, 2].

Exploratory laparoscopy (EL) has prevented 63% of patient's unnecessary laparotomies, which means lower hospital costs secondary to a shortened length of stay [3]. Nowadays, laparoscopy has become an accepted practice in hemodynamically stable patient without signs of peritonitis. We report a case of a lower anterior abdominal gunshot patient with peritoneal penetration treated laparoscopically.

## 2. Case Presentation

Patient's written consent was obtained and any information, including illustrations, was anonymized as far as possible.

A 32-year-old male patient having 180 cm height and 75 kg weight (BMI 23.1) presented to the Emergency Department (ED) of General Hospital of Filiates complaining from a gunshot penetrating injury at left lower anterior abdominal wall.

**Figure 1:**
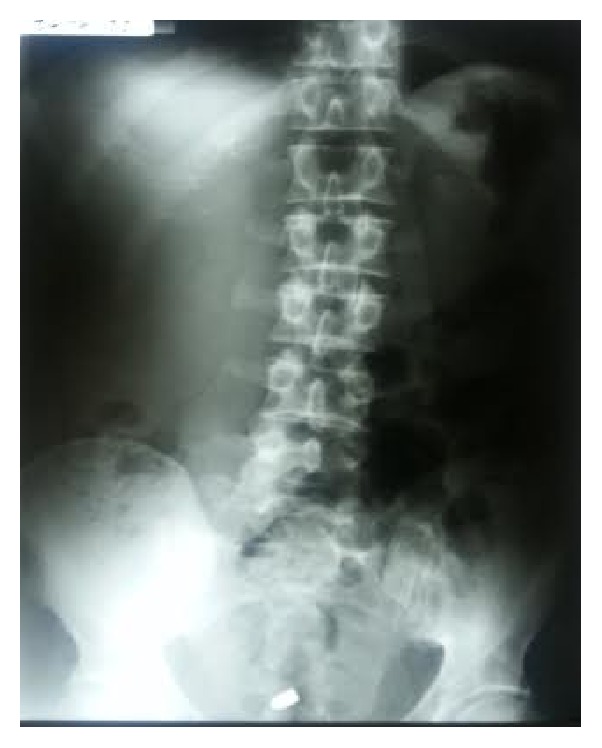
Erect abdominal radiograph shows the bullet in the lower abdomen.

**Figure 2:**
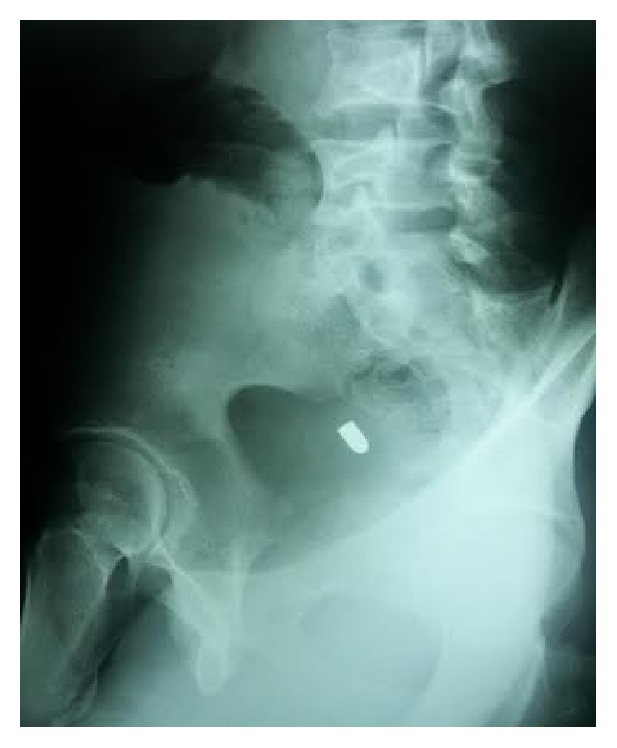
Lateral abdominal radiograph shows the bullet in the pelvic cavity.

**Figure 3:**
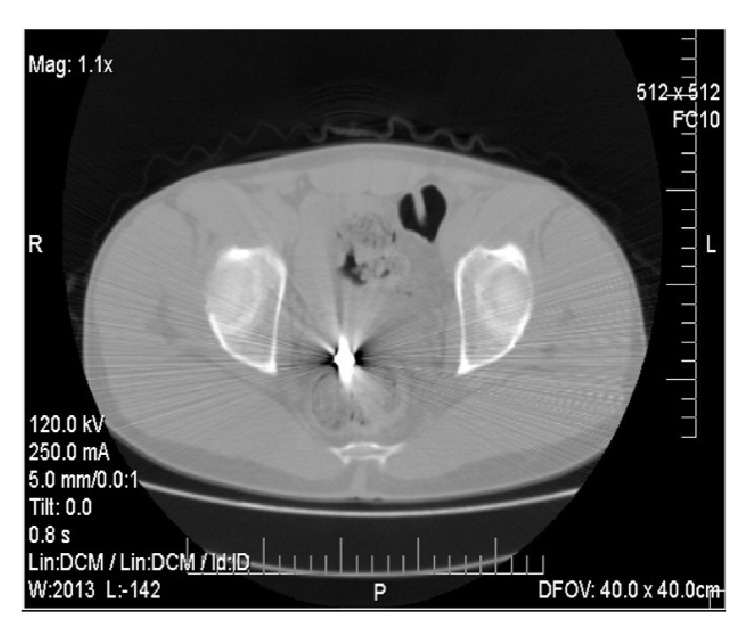
CT scan shows the bullet clear in the lower abdomen.

The patients vital signs were BP 120/80 mm, Hg HR 80/min, and Glasgow Coma Scale (GCS) 15/15. On clinical examination 1 cm diameter entry wound was revealed at the left lower abdominal wall. There was no exit wound. On palpation the abdomen was soft with mild tenderness. The Complete Blood Count (CBC) and urinalysis were normal. Abdominal X-rays showed the bullet in the pelvic cavity (Figures 1 and 2). Chest X-ray did not show free air under the diaphragm. After these examinations, abdominal CT scan was scheduled to identify any serious damage. The CT scan identified the bullet lying in the peritoneal-pelvic cavity and intraperitoneal and retroperitoneal visceral structures were identified to be normal with no injury (Figure 3).

**Figure 4:**
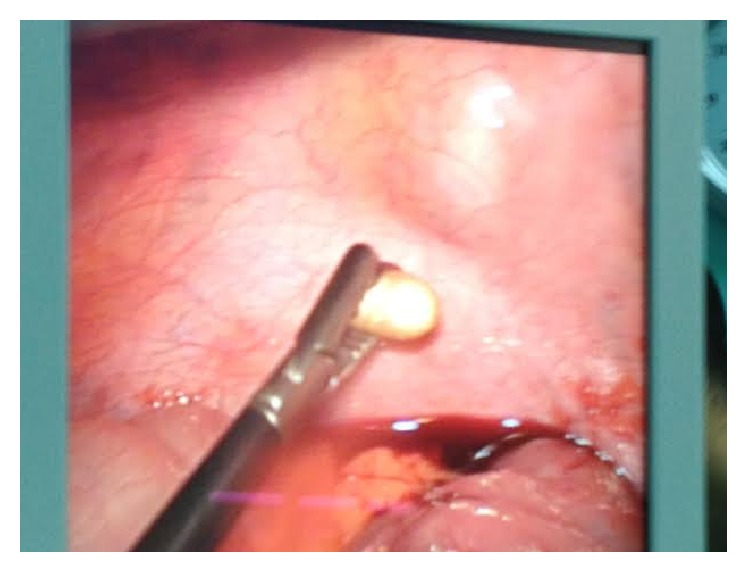
Laparoscopic view of the grasping of the bullet in the Douglas pouch.

After conservative treatment and vital signs follow-up for the first 24 hours and series of clinical examinations, an EL was scheduled. EL identified the bullet in the peritoneal cavity, with no other damages. Finally, the bullet was removed laparoscopically (Figure 4). The patient remained for 24 hours in the surgical department and was discharged from hospital in good conditions.

## 3. Discussion

Penetrating abdominal trauma has been traditionally treated by exploratory laparotomy. As early as 1960, Shaftan was the first who advocated “observant and expectant treatment” rather than laparotomy in penetrating abdominal trauma [4]. The trauma team must recognize the importance of different mechanisms of injury: stab wounds (SWs), gunshot wounds (GSWs), and the velocity of the agent and the different regions of the abdomen (intraperitoneal, retroperitoneal, and thoracoabdominal).

Negative laparotomies result in longer hospital stay (5.3 days when there was no injury association to more than 11 days when associated with injuries) and have an overall higher morbidity and mortality with a higher incidence of complication such as deep vein thrombosis, pulmonary embolism, pneumonia, wound infection, wound dehiscence, abscess formation, and also long term complications such as bowel obstructions from adhesions and ventral hernias [5]. Nowadays, serial physical examination, focus assessment with sonography in trauma (FAST), CT scan, and laparoscopy have increased NOM.

Patients with diffuse abdominal tenderness and hemodynamically unstable should be taken emergently for laparotomy. Hemodynamically stable patient with unreliable clinical examination (i.e., brain injury, spinal cord injury, and intoxication) should have further diagnostic investigation or undergo exploratory laparotomy [6].

Imaging studies should be strongly considered as a diagnostic tool, if the patient is not considered for emergency laparotomy. Velmahos [7] reported the use of CT scan in patients with GSW selected for NOM in 2005. The sensitivity and the specificity of CT scanning were 90.5% and 96%, respectively; in particular a single contrast CT imaging (using IV contrast alone) has a high sensitivity in predicting the need for laparotomy, highest in patients with gunshot wounds. Diagnostic peritoneal lavage (DPL) appears in recent literature to be increasingly replaced from FAST and CT.

Does EL have a position in the context of the management of the trauma patient? The most widely accepted role for EL in the evaluation of the hemodynamically stable patient is to identify peritoneal or diaphragmatic penetration [8, 9]. In our case, there was blood in the abdomen and no active bleeding was recognized. This blood could be derived from the abdominal wall only and does not mandate laparotomy. Laparoscopy is contraindicated in hemodynamic instability, severe traumatic brain injury (intracerebral pressure may be increased by high abdominal pressure), and intra-abdominal adhesions lack of inadequate laparoscopic skills. Tension pneumothorax is always a possible complication while a diaphragmatic injury allows CO_2_ to fill the pleural cavity [10].

**Figure 5:**
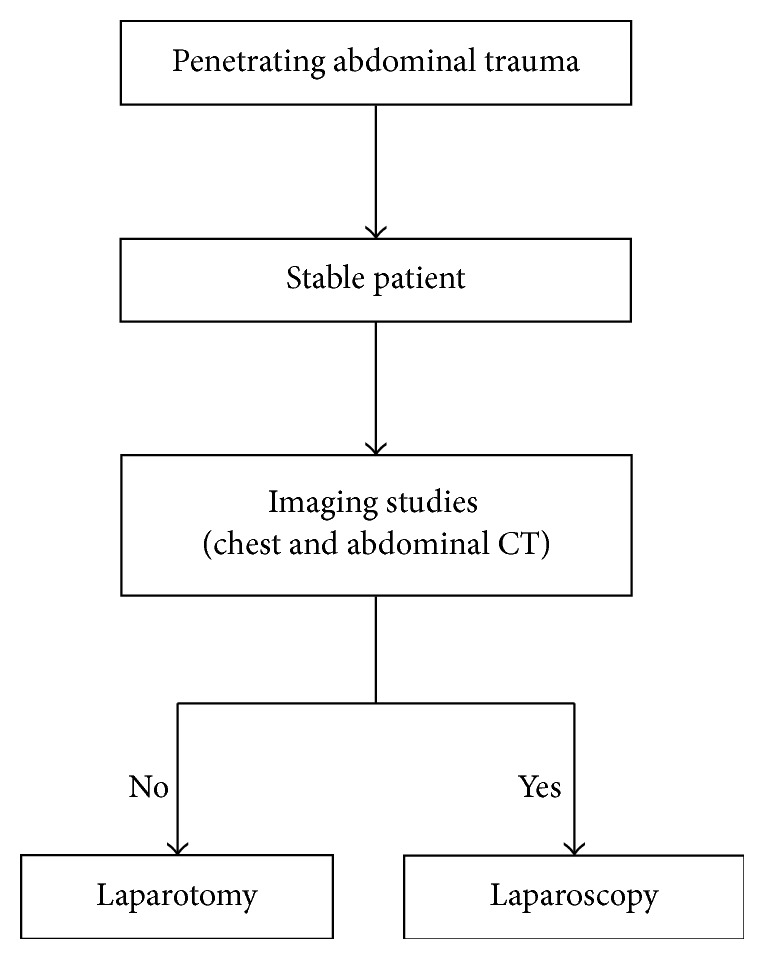
Treatment algorithm. Major criteria were stable patient and no signs of injuries in abdominal viscera.

We report a case with a gunshot entry wound at the left lower anterior abdominal wall, without exit wound. If we estimate the bullet trajectory, the patient should suffer from visceral and vascular injuries. The hemodynamic stability of our patient allowed us to proceed to imaging studies (chest and abdominal X-rays, CT scan, and FAST), which recognized the bullet in the peritoneal-pelvic cavity. All other structures were identified to be normal. We took the decision to do a laparoscopy and remove the bullet (Figure 5). During EL we found the bullet and a small amount of blood within the abdomen, but all viscera appeared unharmed. We removed the bullet and after 24 hours of observation, the patient was discharged from the hospital in good conditions.

The decision for managing gunshot patient should be based on serial reliable clinical examinations, vital signs, and imaging studies. EL is a diagnostic and therapeutic tool, if performed by experienced surgeons, which could prevent patient from unnecessary laparotomy. We believe it represents a safe and feasible method of investigation on a hemodynamic stable patient with anterior abdominal penetrating trauma and no signs of peritonitis.
